# Comparative analysis of the microbiota of sand fly vectors of *Leishmania major* and *L*. *tropica* in a mixed focus of cutaneous leishmaniasis in southeast Tunisia; ecotype shapes the bacterial community structure

**DOI:** 10.1371/journal.pntd.0012458

**Published:** 2024-09-05

**Authors:** Ahmed Tabbabi, Daiki Mizushima, Daisuke S. Yamamoto, Elyes Zhioua, Hirotomo Kato

**Affiliations:** 1 Division of Medical Zoology, Department of Infection and Immunity, Jichi Medical University, Tochigi, Japan; 2 Unit of Vector Ecology, Institut Pasteur de Tunis, Tunis, Tunisia; National Institutes of Health, UNITED STATES OF AMERICA

## Abstract

Phlebotomine sand flies are vectors of the protozoan parasite *Leishmania* spp. Although the intestinal microbiota is involved in a wide range of biological and physiological processes and has the potential to alter vector competence, little is known about the impact of host species and environment on the gut microbiome. To address this issue, a comparative analysis of the microbiota of sand fly vector populations of *Leishmania major* and *L*. *tropica* in a mixed focus of cutaneous leishmaniasis in Tunisia was performed. Bacterial 16S rRNA gene amplification and Illumina MiSeq sequencing were used to characterize and compare the overall bacterial and fungal composition of field-collected sand flies: *Phlebotomus papatasi*, *Ph*. *perniciosus*, *Ph*. *riouxi*, and *Ph*. *sergenti*. Thirty-eight bacterial genera belonging to five phyla were identified in 117 female specimens. The similarities and differences between the microbiome data from different samples collected from three collections were determined using principal coordinate analysis (PCoA). Substantial variations in the bacterial composition were found between geographically distinct populations of the same sand fly species, but not between different species at the same location, suggesting that the microbiota content was structured according to environmental factors rather than host species. These findings suggest that host phylogeny may play a minor role in determining the insect gut microbiota, and its potential to affect the transmission of the *Leishmania* parasite appear to be very low. These results highlight the need for further studies to decode sand fly *Leishmania*-microbiota interactions, as even the same bacterial species, such as *Enterococcus faecalis*, can exert completely opposite effects when confronted with different pathogens within various host insects and vice versa.

## Author summary

*Leishmania major* and *L*. *tropica* are the causative agents of cutaneous leishmaniasis in the Tataouine governorate of southeast Tunisia. In this study, the microbiota of *Phlebotomus papatasi*, *Ph*. *perniciosus*, *Ph*. *riouxi*, and *Ph*. *sergenti*, which were caught from three diverse ecotypes, were analyzed using 16S rRNA gene amplification and Illumina MiSeq sequencing. In total, 117 *Leishmania*-free and blood-non-fed female specimens were used individually in this study to avoid any bias caused by pooling samples. We found that the gut microbiota was mainly controlled by environmental habitats and other factors, as remarkable differences in microbiota composition within the same species collected from the same location was revealed. Moreover, the results suggested that host phylogeny may play a minor role in determining the insect gut microbiota, and the potential of the host factor to affect the transmission of the *Leishmania* parasite appeared to be very low. A better understanding of microorganism-insect-gut bacterial interactions is critical, as the interactions between pathogens and gut microbiota do not adhere to a consistent pattern of synergy or antagonism. Even the same bacterial species, such as *Enterococcus faecalis*, can exert opposing effects when confronted with different pathogens in various host insects

## Introduction

*Leishmania major* and *L*. *tropica* are the causative agents of cutaneous leishmaniasis (CL) in the governorate of Tataouine, southeast Tunisia [1]. CL caused by *L*. *major* has recently appeared as a consequence of vulnerable populations settling in new neighborhoods at the borders of some villages, where the environmental conditions and development of agricultural activities may be favorable for the transmission of *L*. *major*. In contrast, CL cases caused by *L*. *tropica* are scattered and prevail in communities living in villages built on the flanks of the rocky mountains of Tataouine [1].

*Phlebotomus papatasi* is a confirmed vector of *L*. *major* in southwest Asia and North Africa [2,3], including central and southern Tunisia, where reservoir host gerbils are abundant [3,4]. In the recently established settlements mentioned above, *Ph*. *papatasi* is the first species to occupy new homes owing to its specific habits. This species is considered endophilic and can adapt well to anthropogenic environments [5]. However, the spatial distribution of leishmaniasis cases caused by *L*. *tropica* makes it impossible to conclude anthroponotic transmission [1], even though *L*. *tropica* is habitually confirmed to be anthroponotic [6]. The North African gundi (*Ctenodactylus gundi*) is widespread in the mountainous areas of Tataouine and all emerging Tunisian foci of CL caused by *L*. *tropica*, highlighting its potential role as a host reservoir [4,7]. Interestingly, the same *L*. *tropica* genotype was identified in gundis, humans, and *Ph*. *sergenti* within the same southeastern Tunisian *L*. *tropica* foci [1,8–11]. These results suggest the possible existence of two transmission cycles of *L*. *tropica* in this region, with *Ph*. *sergenti* transmitting *L*. *tropica* among humans in and around habitats and transmitting the parasite pathogen among gundis in their mountain habitats. *Ph*. *sergenti* may also be transmitted to humans when they enter areas inhabited by gundi host reservoirs.

Sand flies live in groups and interact with diverse microbiota. Symbiotic interactions can be neutral, harmful, or have beneficial effects on key aspects of insect host fitness, such as development, fecundity, and lifespan [12–15], while the host, in turn, shapes the gut microbiome by changing nutrient availability through diet choice, host metabolism [16], or by triggering immune factors [17]. For example, the nutritive role of the *Proteobacteria* phylum in insect hosts by fixing atmospheric nitrogen has been demonstrated [18]. Similarly, previous studies on *Lutzomyia longipalpis* highlight the importance of bacteria in sand fly growth and development [12]. Conversely, insects tend to mount immune responses to maintain a complex balance between acceptance and rejection, thereby maintaining a peaceful coexistence [17]. The gut bacteria of sand flies can be acquired by their feeding habits (larvae feeding on dead soil organic matter); while, female adults gain blood and sugar meals (plant-fed) through interactions with ingested parasites [19,20]. Indeed, female sand flies become infected when they feed on an infected host and produce a correlative association between the gut microbiota and the ingested parasite, as the growth of *Leishmania* within the sand fly vector occur exclusively in the midgut and hindgut in the presence of symbiotic bacterial communities [20,21]. These microbes play a critical role in *Leishmania* parasite growth, development, and vector competence [20,22–26]. Furthermore, microbes egested during infected sand fly bites augment the severity of leishmaniasis by triggering neutrophil infiltration and facilitating parasitic infection [27]. Most studies have focused on *Leishmania* and its relationships with bacteria and have offered great potential candidates for paratransgenic applications or biological approaches for the control of sand fly populations to prevent *Leishmania* transmission [17,28].

Various factors including the host habitat, diet, developmental stage, and phylogeny affect insect gut microbiota [29–34]. Similar to many other arthropods, next-generation sequencing of the 16S rRNA gene recently revealed complex microbiomes in sand flies. Recently, the number of studies using high-throughput DNA sequencing to investigate the bacterial richness and diversity of sand flies has increased [23,35–40]. Although we appreciate the role of microorganisms and the advent of next-generation sequencing, the factors modulating the composition of the gut microbial community in sand flies are not yet well defined. Using pooled samples, it was recently shown that sand fly gut microbiota is shaped by both host species and geographical location [34,40,41].

In this study, we assessed the microbiota composition of four field-collected sand fly species. The impact of host and environmental variations on the bacterial and fungal contents transported by *Leishmania*-free sand flies was examined in two endemic foci of CL in southeast Tunisia during the period of transmission. Individual samples were used to avoid any bias caused by pooling samples. Tracing the microbiota of wild-caught sand flies will be of considerable benefit in the search for possible vector control candidates for a paratransgenic strategy.

## Materials and methods

### Study area

**Fig 1:**
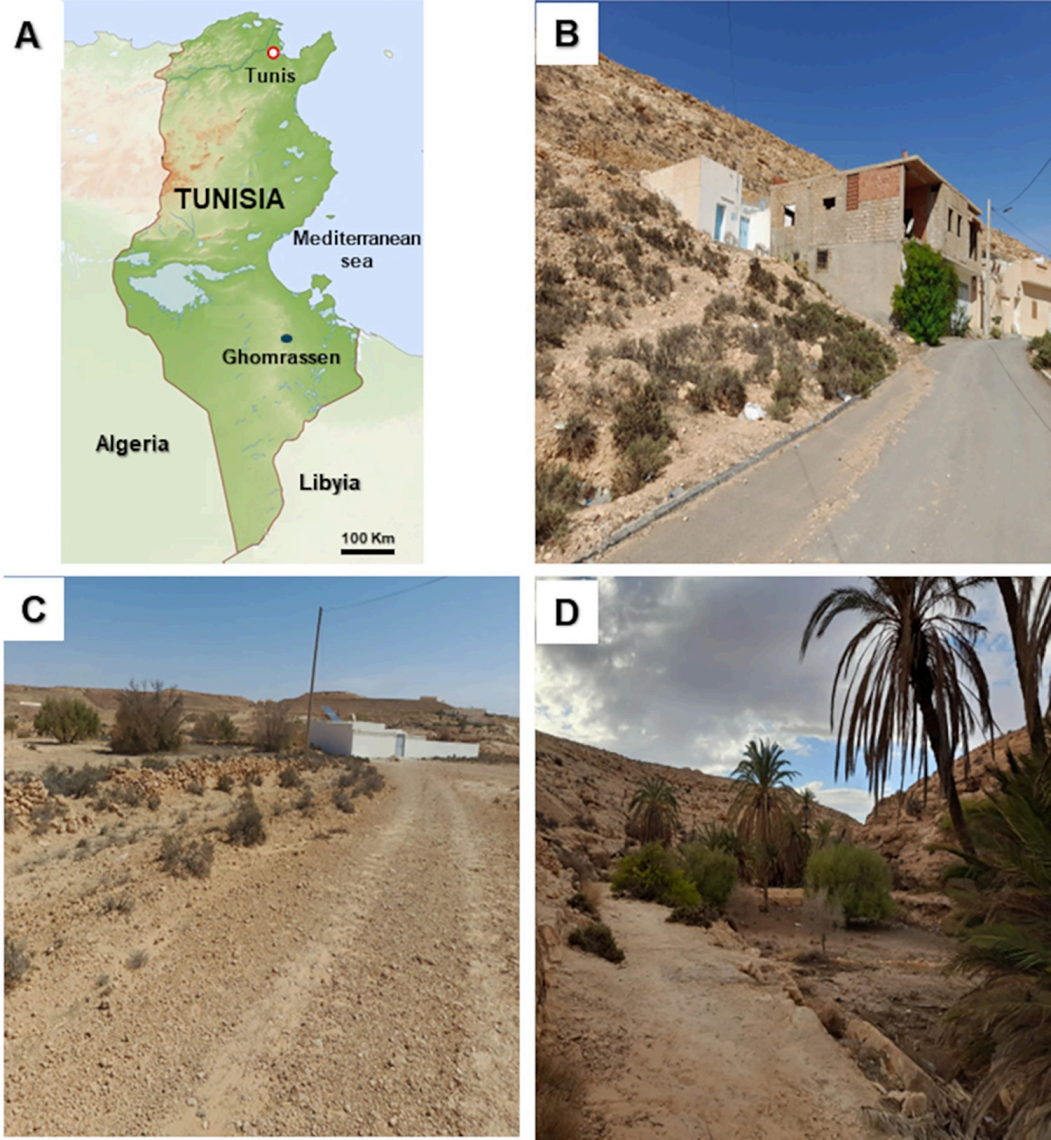
Geographical situation and landscape of sample collection sites. The map of Tunisia on the top left (A) was adapted from a map available at https://www.freeworldmaps.net/africa/tunisia/map.html and shows the geographical situation of the study village, Ghomrassen. The photograph on the top right (B) provides details of the geographical locations of the different sample collection sites: houses of *L*. *tropica*- caused CL patients (white circles), houses of *L*. *major-*caused CL patients (black circles), and *Ctenodactylus gundi* biotopes (white triangle). The photos below represent the houses of *L*. *tropica-*caused CL patients (C), houses of *L*. *major-*caused CL patients (D), and the rocky habitats of *C*. *gundi* (E) (free; no copyright photos).

This study was conducted in the village of Ghomrassen and its borders, an arid bioclimatic area situated in the northern part of the Tataouine governorate in southeast Tunisia (Fig 1). CL caused by *L*. *major* has appeared in new neighborhoods at the borders of some villages [1]. Steppe species dominate sparse vegetation. The area is pastoral with irrigated olive groves and cereals surrounding the water points. However, CL caused by *L*. *tropica* is located in a mountainous area at a moderate altitude (300 m above sea level) [42]. The average annual temperature is 22°C and rainfall fluctuates between 88 and 157 mm (data available from the Tunisian Institute of Meteorology). The regional landscape was determined using basalt-rock escarpments. Gerbils (*Meriones* species) live in the surroundings of the village [11]. The North African gundi, *C*. *gundi*, is the most common wild rodent that inhabits rocky mountain areas [11]. Ghomrassen is a known historical mixed focus of leishmaniasis, where many CL cases caused by *L*. *major* and *L*. *tropica* have been detected in specific households located in new marginal neighborhoods and inside the village, respectively [1].

### Sample collection

Adult sand flies were collected in September 2022 by setting the Centers for Disease Control miniature light traps (John W. Hock Co., Gainesville, FL) at *L*. *major* and *L*. *tropica* sites around the CL cases. Four houses with human CL cases caused by *L*. *major* were situated in new marginal neighborhoods bordered by burrows inhabited by *Meriones* and cultivated vegetation. However, the four houses with human CL cases caused by *L*. *tropica* were built on the stony mountainside bordering the village. Sand flies were collected from animal sheds. In the rocky mountain, the natural ecotype of *C*. *gundi*, traps were placed inside and near the rocky habitats of this wild rodent. The traps were placed 2–3 h before sunset, operated overnight, and retrieved the following morning. Sand flies were washed with distilled water before ethanol fixation to reduce risk contamination. Whole sand fly bodies were subjected to microbiome analysis because we did not have sufficient facilities and equipment to dissect sand flies at the collection sites.

### DNA extraction

Absolute ethanol-fixed sand flies were dried and suspended in 250 μl PBS or saline solution. One gram of 0.1 mm-diameter zirconia/silica beads (BioSpec Products, Bartlesville, OK, USA) was added to the extraction tubes to ease the mechanical crash of microbial cells by the bead crusher μT-12 (Taitec, Saitama, Japan) [43]. Lysed cells were spun down and 200 μL of the supernatant was collected for total DNA isolation. Genomic DNA was purified from each sand fly using the ReliaPrep DNA Clean-up and Concentration System kit (Promega Corporation, Madison, WI, USA) according to the manufacturer’s instructions.

### Identification of sand flies

The collected sand fly specimens were identified by sequence analysis of the mitochondrial cytochrome oxidase I (COI) gene, as previously reported [44]. The sequences were viewed and edited using the BioEdit genetic analyzer. Each sequence was imported into the NCBI Database and nucleotide basic local alignment search tool was used to search for similarity with other sequences in the GenBank using the NCBI database search (https://www.ncbi.nlm.nih.gov/BLAST).

### 16S RNA gene-based identification of bacteria from sand flies by PCR and Illumina MiSeq sequencing

Two PCR steps were performed to amplify the variable region (V3-V4) of the 16S rDNA gene. In the first round, PCR amplification was performed in a thermocycler immediately after DNA extraction, using primers targeting the V3 and V4 regions of bacterial 16S rDNA. The following primers were used to amplify the hypervariable regions V3-V4 of the 16S rDNA gene: forward primer: (5′ TCGTCGGCAGCGTCA GATGTGTATAAGAGACAGCCTACGGGNGGCWGCAG-3′) and reverse primer (5′-GTCTCGTGGGCTCGGAGATGTGTATAAGAGACAGGACTACHVGGGTA TCTAATCC-3′). Two other regions of the 16S rDNA gene (V1-V2 and V4) were used to evaluate the results of some samples obtained by V3-V4 amplification [34,45]. These regions were recommended by the Illumina protocol manual and showed high-quality sequence data, as previously reported [46]. PCR amplification was performed with 35 cycles of denaturation (95°C, 30 sec), annealing (55°C, 30 s), and polymerization (72°C, 30 sec) using AmpliTaq Gold 360 DNA polymerase. Each 1.5 μl portion of the PCR product was re-amplified with the index primers used to generate amplicon libraries for Illumina sequencing [47]. Amplification success was verified on a 1.5% agarose gel. Sequencing was performed using the Illumina MiSeq platform with MiSeq reagent kit version 3 (Illumina, Inc., San Diego, CA, USA). In this context, it is important to note that the main limitation of Illumina is sample loading sensitivity. Overloading the sequencing platform with too much DNA can lead to issues such as overlapping clusters and reduced sequencing quality. Consequently, the overall error rate of this sequencing technology is approximately 1%.

### Detection of *Leishmania* species

To detect *Leishmania* parasites in sand flies, PCR was performed using primers specific for *Leishmania* minicircle kinetoplast DNA [48]. PCR was conducted in a volume of 15 μL using primers and AmpliTaq Gold 360 DNA polymerase. After initial denaturation at 95°C for 10 min, PCR amplification was performed with 35 cycles of denaturation (95°C, 1 min), annealing (55°C, 1 min), and polymerization (72°C, 1 min), followed by a final extension at 72°C for 10 min. The PCR products were analyzed on a 2% agarose gel.

### Quantification of bacteria in sand flies

qPCR with TB Green Fast Mix (Takara Bio Inc., Shiga, Japan) was used to quantify the bacteria in whole sand flies using a Thermal Cycler Dice Real-Time System Lite (Takara Bio Inc., Shiga, Japan). The bacterial 16S rRNA gene was used as the target gene and glyceraldehyde 3-phosphate dehydrogenase (GAPGH) was used as the reference gene. The following primer sequences were used: 5′-ACHCCTACGG GDGGCWGCAG-3′ (16S-q-337F) and 5′-GTDTYACCGCGGYTGCTGGCAC-3′ (16S-q-514R) to amplify the bacterial 16S rRNA gene and 5′- TAATTGGGCTTCTGCTGGGG -3′ (Phgapdh-q-F) and 5′- ACCCTTCAAGTGAGCCGATG -3′ (Phgapdh-q-R) to amplify the GAPDH gene. Relative quantities of 16S rRNA genes were calculated using the ΔΔCt method.

### ITS1-based identification of fungus from sand flies by PCR and Illumina MiSeq sequencing

The same protocol of bacterial DNA amplification, as described above, was used to amplify the fungal ITS1 region. Owing to different types of biases (specificity to fungi, mismatches, length, and taxonomy), various primer combinations targeting different parts of the ITS region were employed jointly, as approved by the Illumina protocol manual [49]. ITS regions were sequenced using the Illumina MiSeq platform and MiSeq reagent kit version 3.

### Data processing and analysis

The reads generated from the MiSeq platform (2 × 301 bp, paired-end format) were produced as fastq files for importation into Quantitative Insights into Microbial Ecology 2 (QIIME2) (version 2020.2.0) [50]. Paired-end reads were trimmed and merged using the DADA2 program in the QIIME2 Plugin. Sequences were clustered into amplicon sequence variants (ASVs) using the QIIME2 program. ASVs were marginalized based on the SILVA version 138 dataset [51], with ≥99% sequence similarity.

Two alpha (abundance-based coverage estimator [ACE] and Pielou), and beta (distance matrix) diversity indices were used to estimate the diversity of sand fly populations in various areas. ACE is an index that reflects bacterial community richness. Pielou index reflects the diversity of bacterial communities. Principal coordinate analysis (PCoA), which is a powerful and popular multivariate analysis method to explore and visualize similarities or dissimilarities in data, was used to display the beta diversity indices of bacterial communities among sand flies [52]. Alpha and beta diversities were analyzed using QIIME2 at a sampling depth of 5055. The group significance of alpha and beta diversity indices was calculated with QIIME2 plugins using the Kruskal–Wallis test and permutational multivariate analysis of variance (PERMANOVA), respectively. Statistical significance was set at p<0.05.

## Results

### Sand fly collection and identification of sand fly species

A total of 117 *Leishmania*-free and blood-unfed females were used individually in this study. Sequences of the mitochondrial COI gene were used to identify the sand fly species. Four sand fly species including 85 *Ph*. *papatasi*, 15 *Ph*. *perniciosus*, 5 *Ph*. *riouxi*, and 12 *Ph*. *sergenti* were identified.

### Microbiome profile of field-collected sand flies

**Fig 2:**
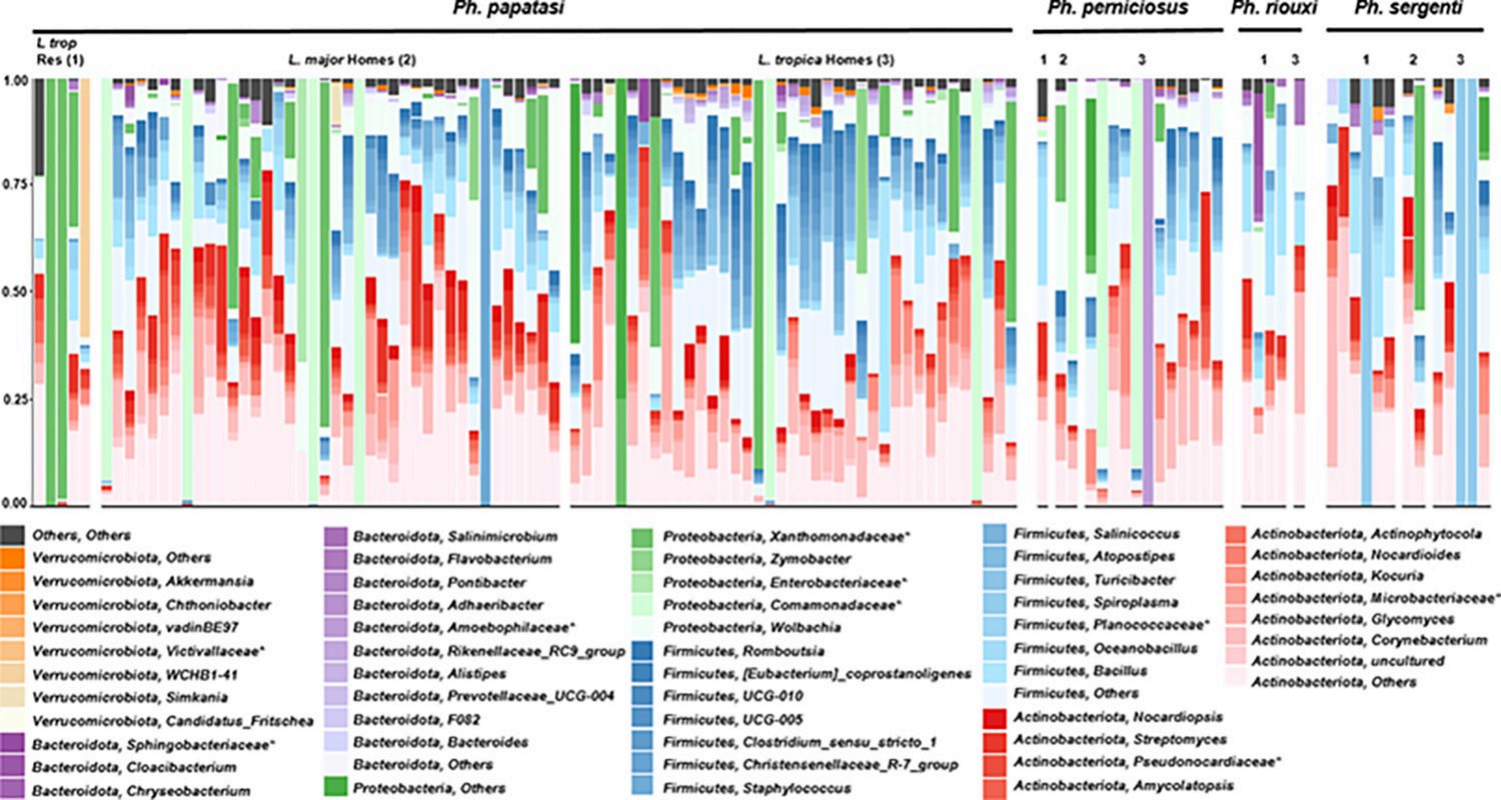
Bacterial genus composition of different sand fly species: *Ph*. *papatasi*, *Ph*. *perniciosus*, *Ph*. *riouxi*, and *Ph*. *sergenti*. The asterisk indicates other-level classifications when Qiime2 failed to provide the genus level.

High-throughput sequencing of the 16S rRNA gene was performed to investigate the bacterial diversity of four adult sand fly species caught from three different locations. Taxonomic compositions were compared in the same sand fly species at different sampling locations, and in different sand fly species from the same sampling locations at the phylum and genus levels. Thirty-eight genera belonging to five phyla (*Actinobacteriota*, *Firmicutes*, *Proteobacteria*, *Bacteroidota*, and *Verrucomicrobiota*) were identified, with a few unclassified taxa at different ranks (Fig 2). *Actinobacteriota* and *Firmicutes* were the most prevalent (39.1% and 36.3%, respectively) phyla. Among the 38 bacterial genera, 18 have never been reported in field-collected sand flies (*Akkermansia*, Chthoniobacter, *Simkania*, *Candidatus fritschea*, *Cloacibacterium*, *Salinimicrobium*, *Pontibacter*, *Adhaeribacter*, *Alistipes*, *Bacteroides*, *Romboutsia*, *Coprostanoligenes*, *Salinicoccus*, *Atopostipes*, *Turicibacter*, *Streptomyces*, *Amycolatopsis*, *and Actinophytocola*). Overall, twenty-two bacterial genera contained pathogenic species for humans (46.4%). In the case of *Ph*. *papatasi*, the pathogenic bacteria were slightly more abundant in *L*. *major* homes (47.3%) than in *L*. *tropica* homes (39.7%).

### Impact of environmental habitat on microbiome structure

**Fig 3:**
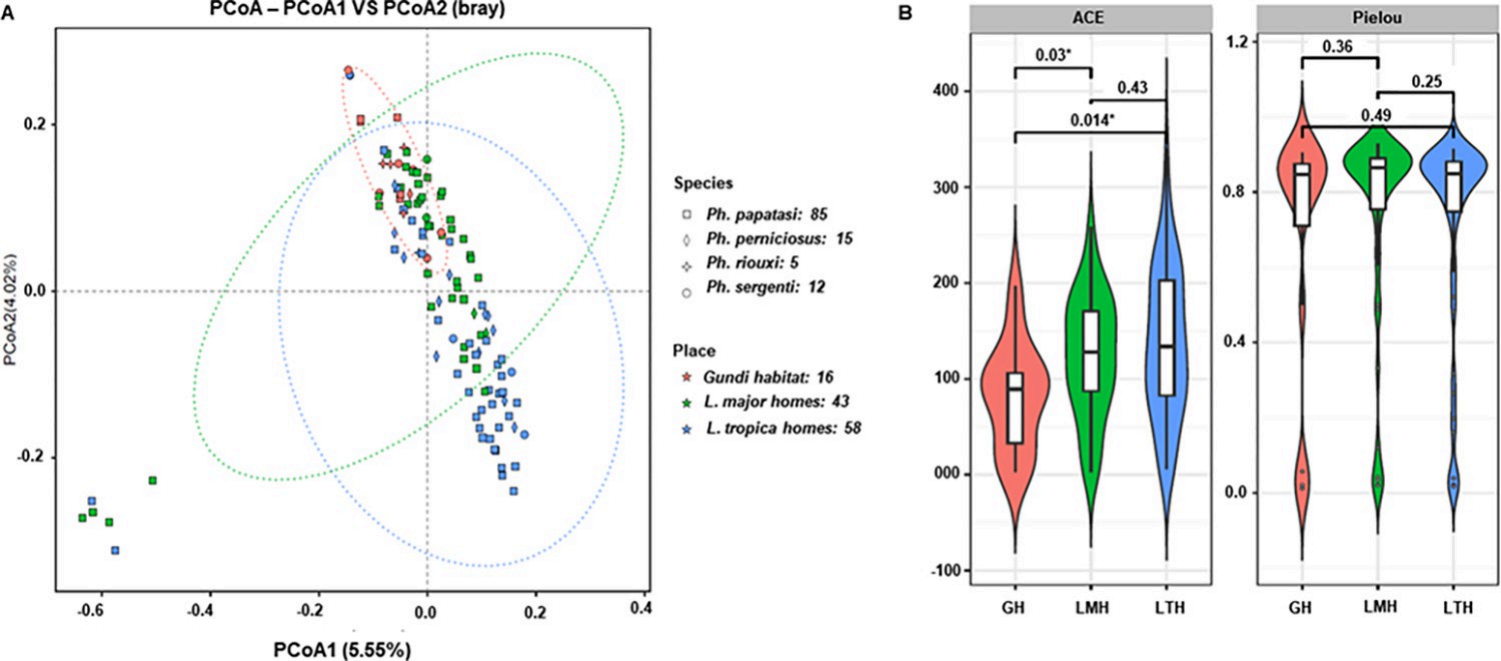
**a)** Bacterial PCoA plot illustrating beta diversity distance matrices of the Bray–Curtis distance comparing the sample distribution among the three locations. **b)** Bacterial box plots of the alpha diversity measure using ACE and Pielou diversity indices at the three locations. GH: Gundi habitat; LMH: *L*. *major* homes; LTH: *L*. *tropica* homes.

To study the effect of the ecological habitat of sand flies on the gut bacterial community, we compared two alpha (ACE and Pielou) and beta (distance matrix) diversity indices of the microbial community of each sand fly species at different sampling locations (16 flies in the gundi habitat, 43 flies in zoonotic CL [ZCL] homes, and 58 flies in ACL homes). The similarities and differences between the microbiome data from different samples collected from the three collections were explored and visualized using principal coordinate analysis (PCoA). As shown in Fig 3A, the PCoA plot revealed three different sample groups based on the environmental habitat, indicating that the microbial structures from the same area were close to each other, whereas those from different locations were discrete (PERMANOVA, F = 2.44, p<0.05). The observed pattern strongly suggests that all sand flies collected from different sites harbored distinct microbiota, highlighting a strong correlation between the sand fly microbiota association and the environment in which they reside. In this context, it is important to note that the bacterial composition of some samples appeared completely different from others, and further investigation is needed to confirm these results. Different primers were used to amplify bacterial DNA from the same sand fly sample and similar results were obtained (S1 Fig). Moreover, bacterial community richness, as measured by the ACE index, was significantly higher in *L*. *major* and *L*. *tropica* homes than in the gundi habitat (p<0.05), which may have been due to exposure to several bacterial sources (Fig 3B). In the case of *Ph*. *papatasi*, *Actinobacteriota* (mean: 44.3%; median: 41.3%) was more dominant bacterial genus in *L*. *major* homes than in *L*. *tropica* homes (mean: 33.2%; median: 28.5%) (Exact Wilcoxon rank sum test, W = 590, p = 0.06), while *Firmicutes* was the most frequently isolated from sand flies collected in *L*. *tropica* homes (mean: 37.3%; median: 37.6%) compared to *L*. *major* homes (mean: 28.1%; median: 27.2%) (Exact Wilcoxon rank sum test, W = 999, p = 0.03) (Figs 2 and S2). Overlap was clearly observed between microbes isolated from different areas, including a number of soil and environmental organisms, such as *Corynebacterium* species.

### Impact of host species on microbiome structure

To study the effect of host species on the gut bacterial community, we compared the beta (distance matrix) diversity indices of the microbial communities of different host species from the same sampling locations. The similarities and differences between the microbiome data from different samples were explored and visualized using PCoA (Figs 2 and S3). The PCoA plot showed an overlap in the bacterial contents of 85 *Ph*. *papatasi*, 15 *Ph*. *perniciosus*, 5 *Ph*. *riouxi* (small sample size), and 12 *Ph*. *sergenti* (PERMANOVA, *F* = 1.5, p<0.05). This pattern suggested that all sand fly species harbored similar microbes, indicating that variations in the sand fly gut microbiota are not expressed by host genetic factors.

### Fungal profile of field-collected sand flies

**Fig 4:**
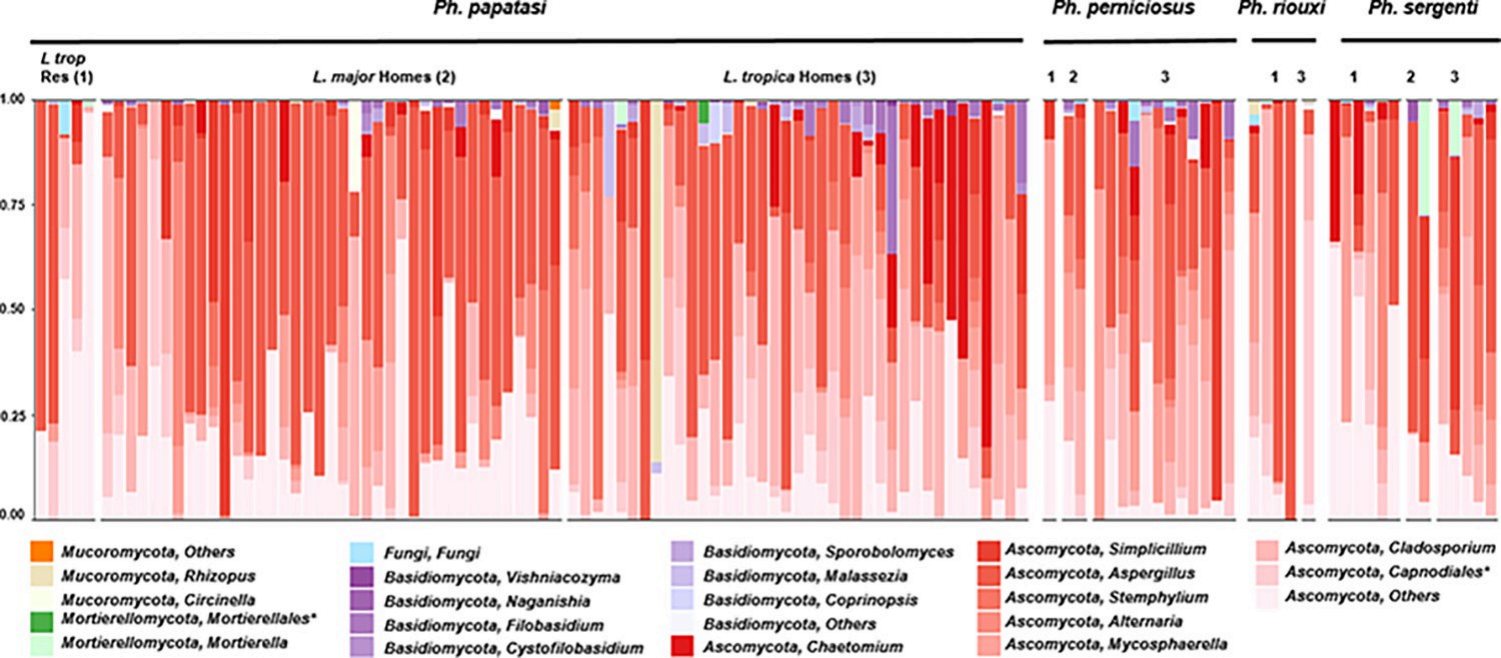
Fungal genus composition of different sand fly species: *Ph*. *papatasi*, *Ph*. *perniciosus*, *Ph*. *riouxi*, and *Ph*. *sergenti*. The asterisk indicates other-level classifications when Qiime2 failed to provide the genus level.

We characterized the fungal profile*s* of the same sand fly species collected from different sampling locations and of different host species from the same sampling locations. We identified a higher diversity than previously reported [39] (17 different genera belonging to 4 phyla), with a few unclassified taxa at different ranks (Fig 4). Among these, 10 genera were not associated with phlebotomines (*Circinella*, *Vishniacozyma*, *Naganishia*, *Filobasidium*, *Cystofilobasidium*, *Sporobolomyces*, *Coprinopsis*, *Stemphylium*, *Alternaria*, and *Mycosphaerella*). *Chaetomium* genus was recently identified in larvae reared on sand flies. *Ascomycota* phylum was more abundant than other genera (97.2%).

### Impact of environmental habitat on fungal community

**Fig 5:**
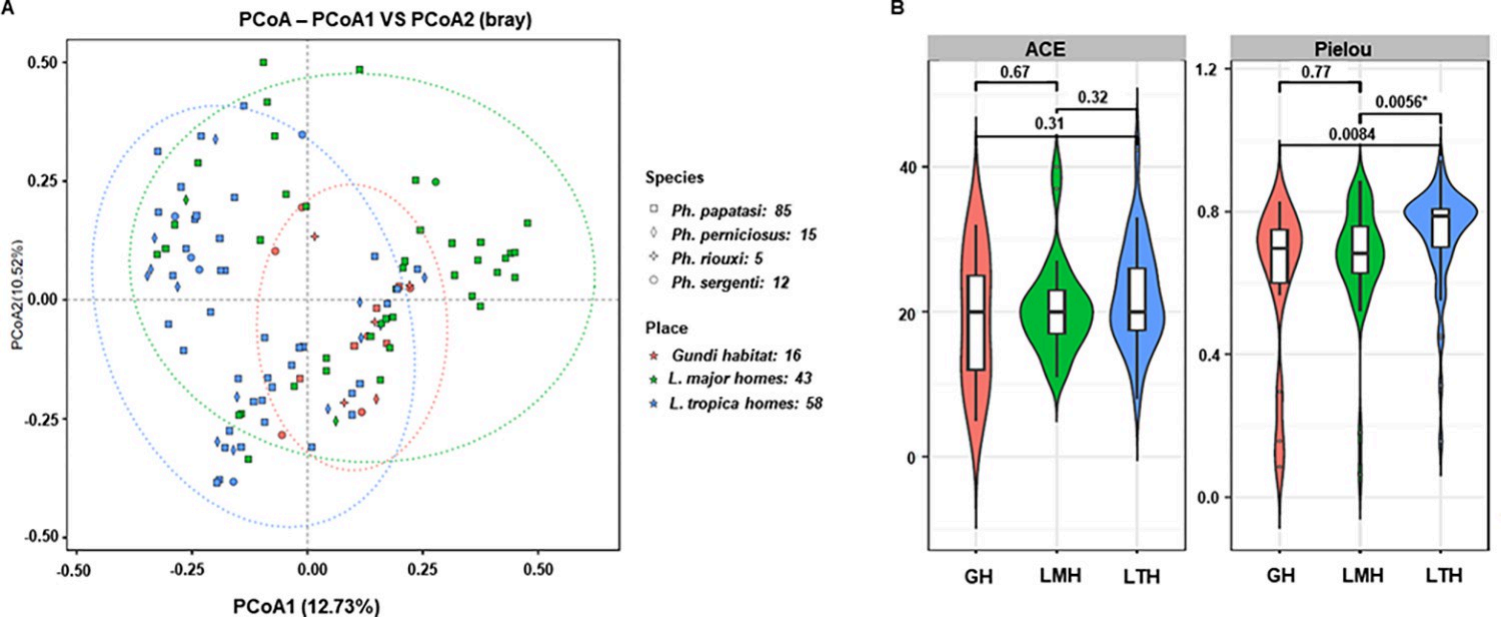
**a)** Fungal PCoA plot illustrating beta diversity distance matrices of the Bray–Curtis distance comparing the sample distribution among the three locations. **b**) Fungal box plots of the alpha diversity measure using ACE and Pielou diversity indices at the three locations. GH: Gundi habitat; LMH: *L*. *major* homes; LTH: *L*. *tropica* homes.

The fungal structure of sand fly species collected from the three collections was visualized using PCoA (Fig 5A), which showed that the environmental habitat shapes the fungal communities, as reported above, with bacteria (PERMANOVA, *F* = 5.5, p<0.05). Moreover, no difference in fungal community richness (ACE) was detected among the three locations; however, *L*. *tropica* homes showed the highest evenness of species distribution, as measured by the Pielou evenness index, compared with 2 other habitats (p<0.05) (Fig 5B). In the case of *Ph*. *papatasi*, *Aspergillus* (41.0%) was the most dominant fungal genus in *L*. *major* homes compared to *L*. *tropica* homes (24.5%), whereas *Cladosporium* was the most frequently isolated genus from sand flies collected in *L*. *tropica* homes (26.3%) compared to *L*. *major* homes (11.1%) (Fig 4).

### Impact of host species on fungal community

The PCoA plot showed an overlap of the fungal communities of 85 *Ph*. *papatasi*, 15 *Ph*. *perniciosus*, 5 *Ph*. *riouxi* (small sample size limitation), and 12 *Ph*. *sergenti*, indicating that host species factors did not influence the overall composition of the fungal structure, as reported above, with bacteria (PERMANOVA, *F* = 1.6, p = 0.05) (S4 Fig).

### Bacterial quantification

Any manipulation that reduces the size/diversity of natural microbiota affects the ability of *Leishmania* to establish infections in sand flies [24]. The relative quantity of the bacterial communities was assessed in *Ph*. *papatsi* at different sampling locations. Our results showed that the bacteria differed slightly between the sand fly ecotypes (Gundi habitat: 0.0007; *L*. *major* homes: 0.001; *L*. *tropica* homes: 0.0008); however, it was not possible to conclude clear outcomes regarding a quantitative interaction with *Leishmania* transmission areas (S5 Fig).

## Discussion

Microbiota communities were determined in sand fly populations in a mixed focus of CL caused by *L*. *major* and *L*. *tropica* in Tunisia, and the effects of host species and environmental habitats on the bacterial structure were explored. Because of the difficulties associated with obtaining only midgut samples, as the present study was carried out for vector research and the collected sand flies had to be identified at the species level, whole sand fly bodies from different areas in Tunisia were employed in the microbiome analysis. Our findings revealed that *Actinobacteria* (39.1%), *Firmicutes* (36.3%), and *Proteobacteria* (19.5%) were the main phyla in the four adult sand fly species caught from three different locations. These results are partly in accordance with previous data illustrated by a meta-analysis of the respective gut microbiota of Old and New World sand flies, which showed that more than 57% of the identified bacteria belonged to the *Proteobacteria* phylum in *Lutzomyia* sp., whereas for Old World sand flies, *Proteobacteria* (47.0%) and *Firmicutes* (40.0%) were preponderant [23]. Bacteria of the *Actinobacteria* phylum account for 12.7% and 11.9% of *Lutzomyia* and *Phlebotomus* sand flies, respectively [23]. *Actinobacteria* defend ants, beetles, and wasps against detrimental microorganisms by producing antibiotics. The remarkable capacity of *Actinobacteria* to exploit a wide variety of carbon and nitrogen sources and their extensive repertoire of secondary metabolites predisposes this group to engage in protective symbioses [53]. Defensive mutualism with *Actinobacteria* may represent a general and widespread phenomenon in arthropod biology, ecology, and evolution.

The 117 studied samples were screened for *Leishmania* DNA and no positivity was detected. These findings were not surprising, because the rate of *Leishmania* infection in sand flies is estimated to be very low in nature, even in endemic areas [54]. The resident microbiota of sand flies has a strong effect on vector competence and can partly explain the origin of these results [20,38]. Among the bacterial genera associated with the sand fly midgut, a significant number of bacterial genera known to contain pathogenic species were recorded in the sand fly vectors of *L*. *major* and *L*. *tropica*, suggesting that sand flies may cause additional health concerns to humans and livestock. However, further studies are needed to determine the risk of these pathogenic bacteria, as their presence in the midgut is insufficient to indicate that sand flies are possible gut bacterial vectors. Furthermore, whether certain clinical outcomes of leishmaniasis are linked to bacteria potentially deposited during *Leishmania*-infected sand fly bites remains to be fully understood [55]. The relative prevalence of putative pathogens was slightly dependent on location, and there was no significant correlation between the habitat and prevalence of pathogenic bacteria. *Ph*. *papatasi*, which is the main ZCL vector in *L*. *major*, carries a different rate of pathogenic bacteria than the same species in *L*. *tropica*, in which there is no *L*. *major* transmission. Notably, in this study, alterations in the gut microbiota of *Ph*. *sergenti* were not clear due to a short collection period and density rarity. Extending the collection period may completely change the bacterial structure owing to seasonal variations [23].

Differences in the richness and diversity of gut bacterial composition were found between sand flies belonging to two distinct ZCL and CCL ecotypes, as well as between sand fly species. These findings suggest that the structural shape of the associated microbiota may influence susceptibility to *Leishmania* infection in both foci; however, it was not possible to elucidate their role or conclude clear outcomes regarding potential pathogenic effects or interactions with *Leishmania*. Any manipulation that reduces the richness/diversity of the natural microbiota affects the ability of *Leishmania* to establish infections in sand flies [20,38]. Previous studies have shown that treatment with antibiotics reduces the richness and diversity of the microbiota but increases *Leishmania* infection, indicating that the microbiota can be a barrier to the establishment and development of promastigotes in *Ph*. *papatasi* and *Pintomyia evansi* [38,56]. In contrast, antibiotic-mediated perturbation of the midgut microbiome in *Ph*. *dubosqui* and *Lu*. *longipalpis* cannot support *Leishmania* growth [20,24]. Collectively, these data suggest that the sand fly midgut microbiome is a critical factor for *Leishmania* growth and differentiation before disease transmission. It is essential to determine the bacterial species and mechanism(s) by which the bacterial microbiome may enhance or repress *Leishmania* development in the sand fly gut. Conversely, it has been demonstrated that pathogens such as *Plasmodium falciparum*, Zika, and Chikungunya viruses can shape the abundance and composition of the mosquito gut microbiome [57–60]. On the other hand, our results showed that the bacterial load changed slightly among the three different *Ph*. *papatasi* populations, suggesting a quantitative interaction with *Leishmania* parasites.

Although it is generally accepted that host species and ecological factors can have a strong impact on the gut microbiota of insects [30,61,62], little is known about the impact of these factors on sand fly microbiota. Substantial variations in gut bacterial composition were found between geographically distinct populations of the same sand fly species in the present study, suggesting that the sand fly gut microbiota is shaped by geographical location, potentially affecting pathogen acquisition and transmission by sand fly vectors. These findings highlight the need for further studies to decode the roles of environmental factors in determining the gut microbiome and, therefore, the susceptibility to *Leishmania* infection in sand fly vectors. However, the identification of the same bacterial composition in different host species from the same sampling location suggests from one side that host phylogeny may play a minor role in determining the insect gut microbiota, and its potential to affect the transmission of the *Leishmania* parasite appears to be very low. In this context, it is important to mentioned that due to the limitations of field sampling, sand flies were washed with distilled water and fixed with absolute ethanol, and then whole bodies were used for DNA extraction. The washing protocol is not exactly the same as in our previous study [39,40]. Therefore, a risk of contamination with bacteria on the body surface may be considered and may have an impact on the correlation between sand fly microbiota and their environment. Further studies using only midgut samples are needed to clarify this issue. Moreover, no inter-population genetic variations were observed, indicating the absence of environmental barriers between the three studied locations. A better understanding of the sand fly *Leishmania*-microbiota interactions is critical, as it is possible that even the same bacterial species can exert completely opposite effects when confronted with different pathogens within various host insects, and vice versa. However, some extrinsic and intrinsic factors (cited below) may bias this hypothesis. According to a phylosymbiosis study, closely related species often host similar microbiota [63]. It has recently been demonstrated in laboratory-colonized sand flies that phylogenetically closely related species have similar compositions of their associated microbial communities, which may explain the obtained results, as the four species belonged to the same *Phlebotomus* genus [40]. Moreover, the host taxa in these studies were mainly across genera and not at species level, limiting precise data analysis [34,40,41]. Recently, it has been shown that host phylogeny is a strong driver of microbiota structure in sand flies [34,40]. Another study showed that both environmental and host species factors can have a marked effect on microbial communities in the sand fly midgut, which is in agreement with our results [41]. Importantly, contrary to previously cited studies, we used individual samples to avoid any biased results, and a remarkable difference in microbiota structure within the same species collected from the same locations revealed a large individual variation in bacterial composition. Several factors are known to affect the structure of microbial communities, including host species, population genetic variability, blood-meal origin, larval and adult habitat, climate, temperature, humidity, location and period of collection, body size, sex, stage of life cycle, co-infection with pathogens and microorganisms, and resistance to insecticides [23,30,61–63,64–74].

In this study, we identified the *Bacillus* genus in *Ph*. *papatasi* and *Ph*. *perniciosus*, as expected, since the bacteria of this genus are found in almost all Old World sand fly species [23]. A recent meta-analysis of sand fly-associated bacteria showed that bacteria belonging to the *Bacillus* genus exhibit a host-specific distribution, with only *Bacillus subtilis* isolated from more than one sand fly species (*Ph*. *halepensis*, *Ph*. *papatasi*, and *Ph*. *perniciosus*) [23]. Its occurrence in several sand fly species and its nonpathogenic, easily culturable, and genetically malleable nature make it a potential candidate for paratransgenic or biological approaches for the control of sand fly populations to prevent *Leishmania* transmission. It has previously been used as a promising paratransgenic agent to impair parasite growth and reduce *Leishmania* transmission [23,75,76]. In addition to the *Bacillus* genus, we identified the *Wolbachia* genus from *Ph*. *papatasi* and *Ph*. *perniciosus*, which are known to be suitable paratransgenic agents and are used to develop paratransgenic mosquitoes [77–81]. Studies on sand flies have shown high infection rates of *Wolbachia* by *Ph*. *papatasi* and *Ph*. *perniciosus* (81.7% and 60.3%, respectively), but the impact of *Wolbachia* on *Leishmania* infection load remains unclear [82].

Only a few studies have attempted to identify fungal diversity in sand flies [39,83–85] and to the best of our knowledge, this is the first study to show the impact of host species and ecological factors on insect gut fungi. As reported above for bacteria, substantial variations in the gut fungal composition were found between geographically distinct populations of the same sand fly species, suggesting that the sand fly gut fungus is shaped by the environmental habitat, and the host phylogeny probably plays a minor role in determining insect gut fungi, and therefore, in susceptibility to *Leishmania* infection. However, it has been demonstrated that fungi can modulate vector competence in mosquitoes [86]. Among the detected fungi, 10 genera were not associated with phlebotomines, which expands our knowledge of their occurrence in sand flies. Ascomycetes, including *Aspergillus* and *Cladosporium* are opportunistic pathogens found in humans and other animal hosts [87]. These two genera were dominant in *L*. *major* and *L*. *tropica* homes. This suggests that sand flies may pose an additional threat to human and livestock health. However, the risk posed by these pathogenic fungi remains unclear. Interestingly, *Malassezia* genus was present in all sand fly species, which may pave the way for the functional characterization of sand fly-associated fungi. A previous study reported a relationship between *L*. *infantum* infection in dogs without skin lesions and increased growth of *Malassezia pachydermatis* with low phospholipase activity [88].

## Conclusions

This study describes the complex microbial community structure in sand fly vectors of *L*. *major* and *L*. *tropica* with a mixed focus on CL in Tunisia. Our results showed that the microbiota content was structured according to environmental factors rather than the host species. Sand flies have adapted to survive in diverse habitats, feed on the blood of a wide range of hosts, and transmit various *Leishmania* species. Our findings suggest that the ecological diversity of sand flies may contribute to shaping the structure of the associated microbiota. However, the revelation of the same bacterial composition in different host species from the same sampling location suggests that host phylogeny may play a minor role in determining the insect gut microbiota and its potential to affect the transmission of the *Leishmania* parasite appears to be very low.

## Supporting information

S1 Data Excel spreadsheet containing, in separate sheets, the underlying numerical data and statistical analysis for Figs 2, 3A, 3B, S2, and S3. (XLSX)

S2 Data Excel spreadsheet containing, in separate sheets, the underlying numerical data and statistical analysis for Figs 4, 5A, 5B, and S4. (XLSX)

S3 Data Excel spreadsheet containing, in separate sheets, the underlying numerical data for S1 Fig. (XLSX)

S4 Data Excel spreadsheet containing, in separate sheets, the underlying numerical data for S5 Fig. (XLSX)

S1 Fig Bacterial genus composition of sand fly samples using different primers. The asterisk indicates other-level classifications when Qiime2 failed to provide the genus level.
(TIF)

S2 Fig Percentages of *Actinobacteriota* and *Firmicutes* in *Ph*. *papatasi* collected from *L*. *major* and *L*. *tropica* homes. The line indicates the median, and the lozenge indicates the mean. GH: Gundi habitat; LMH: *L*. *major* homes; LTH: *L*. *tropica* homes.
(TIF)

S3 Fig Bacterial PCoA plot illustrating beta diversity distance matrices of the Bray–Curtis distance compared with the sample distribution among the four species. (TIF)

S4 Fig Fungal PCoA plot illustrating beta diversity distance matrices of the Bray–Curtis distance compared with the sample distribution among the four species. (TIF)

S5 Fig Relative quantities of *Ph*. *papatasi* collected from three locations. GH: Gundi habitat; LMH: *L*. *major* homes; LTH: *L*. *tropica* homes. (TIF)
