# Supplementary figures and images for: Comparative analysis of the microbiota of sand fly vectors of Leishmania major and L. tropica in a mixed focus of cutaneous leishmaniasis in southeast Tunisia; ecotype shapes the bacterial community structure
Source: PLoS Negl Trop Dis. 2024 Sep 5;18(9):e0012458. doi: 10.1371/journal.pntd.0012458 (PMC11407667; doi:10.1371/journal.pntd.0012458)

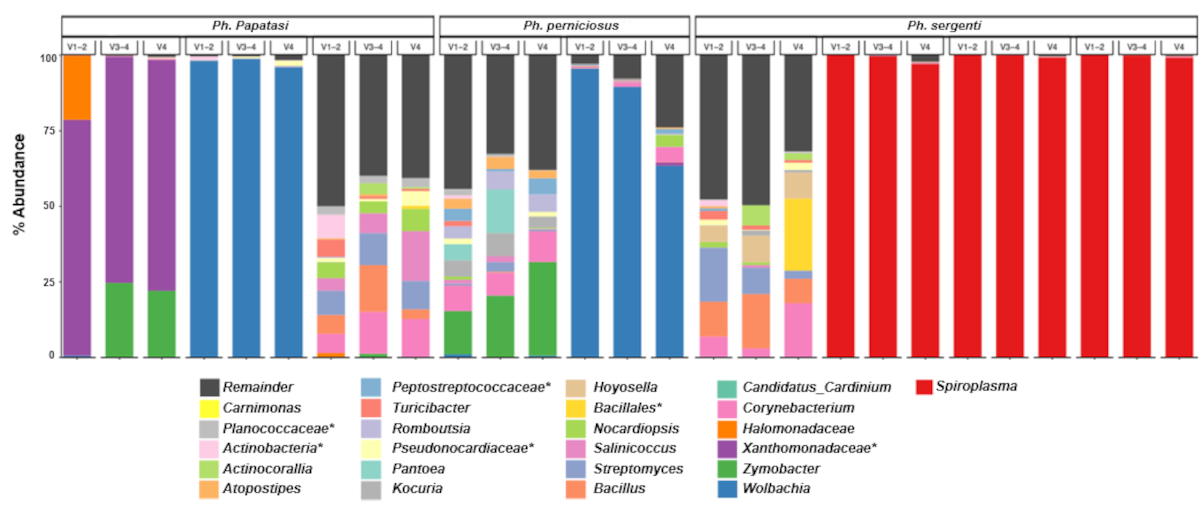

Supplement: S1 Fig — The asterisk indicates other-level classifications when Qiime2 failed to provide the genus level. (TIF) [file pntd.0012458.s005.tif]

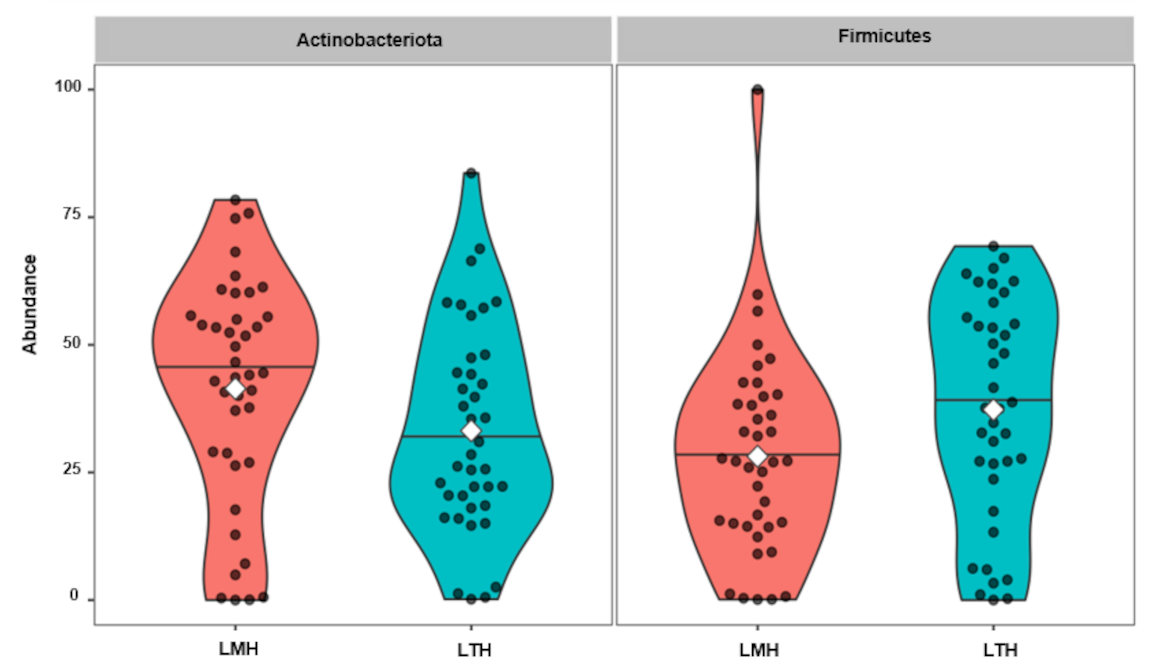

Supplement: S2 Fig — GH: Gundi habitat; LMH: L. major homes; LTH: L. tropica homes. (TIF) [file pntd.0012458.s006.tif]

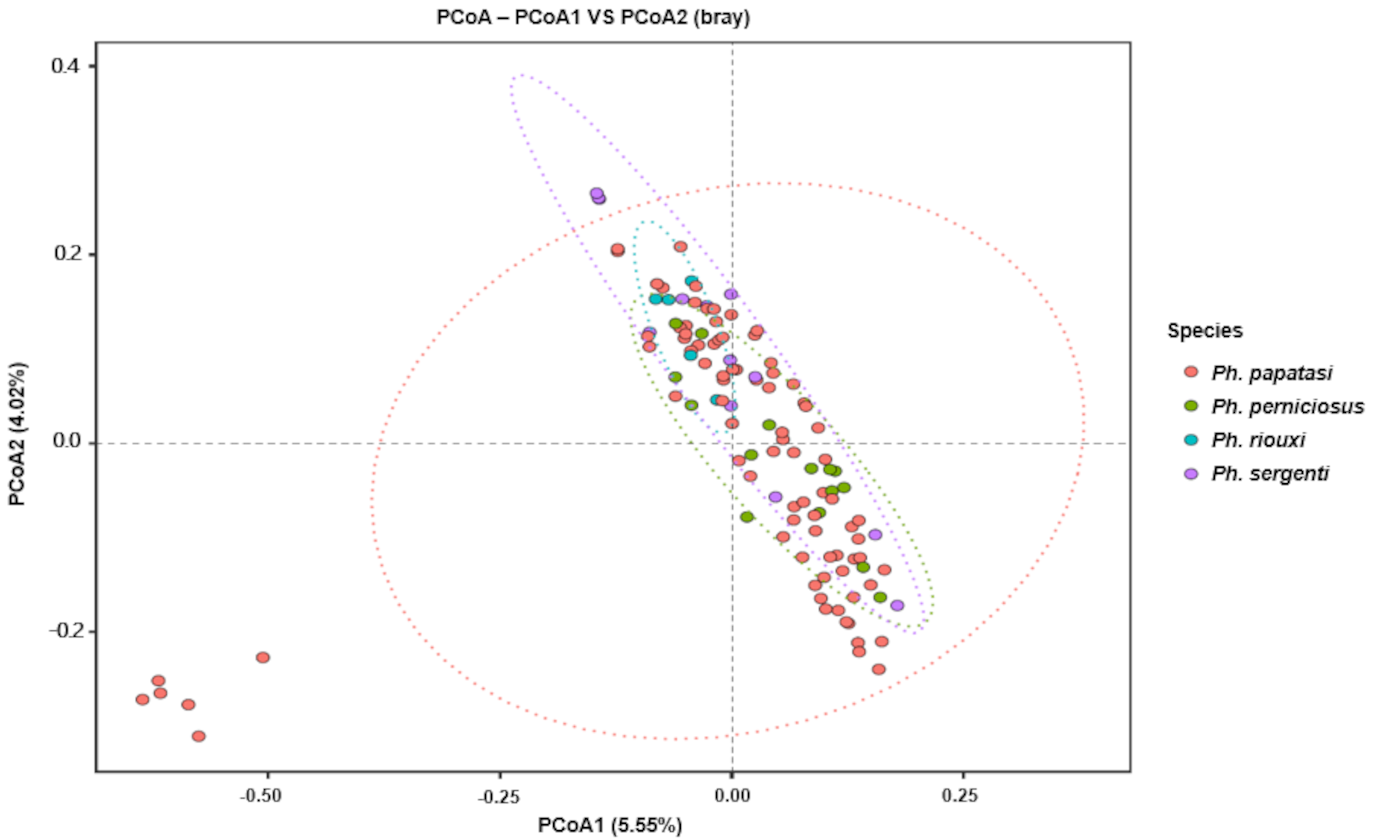

Supplement: S3 Fig — (TIF) [file pntd.0012458.s007.tif]

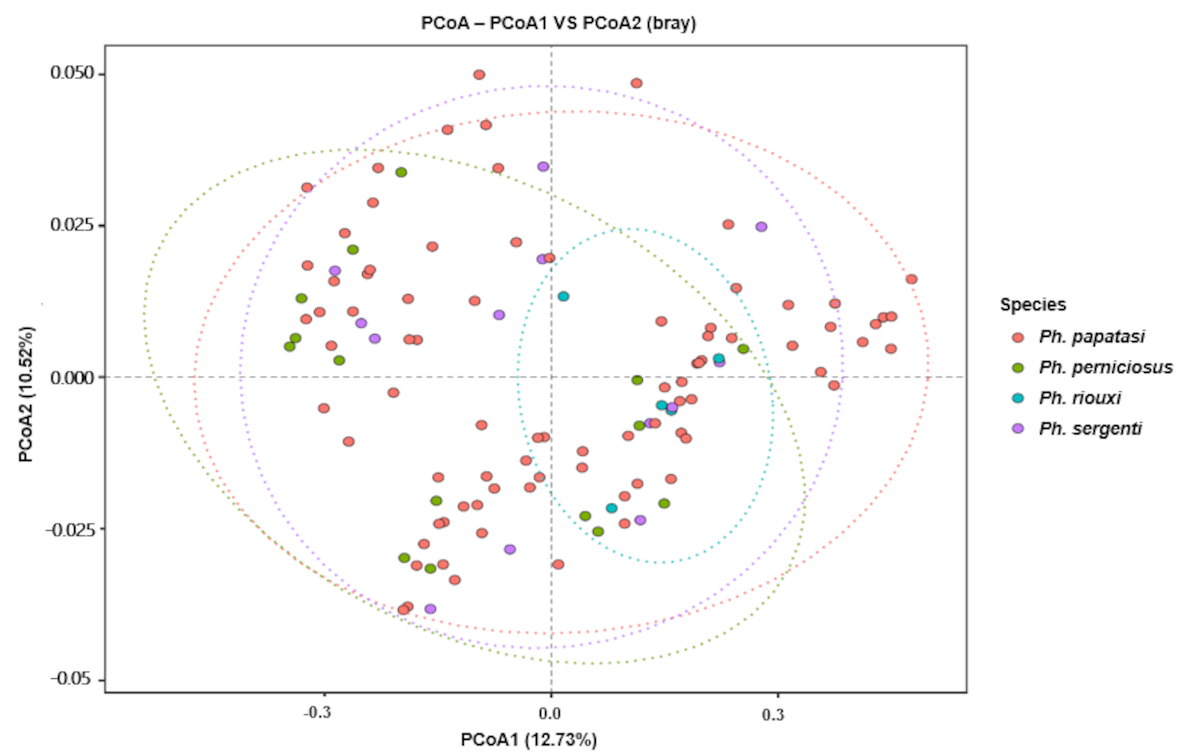

Supplement: S4 Fig — (TIF) [file pntd.0012458.s008.tif]

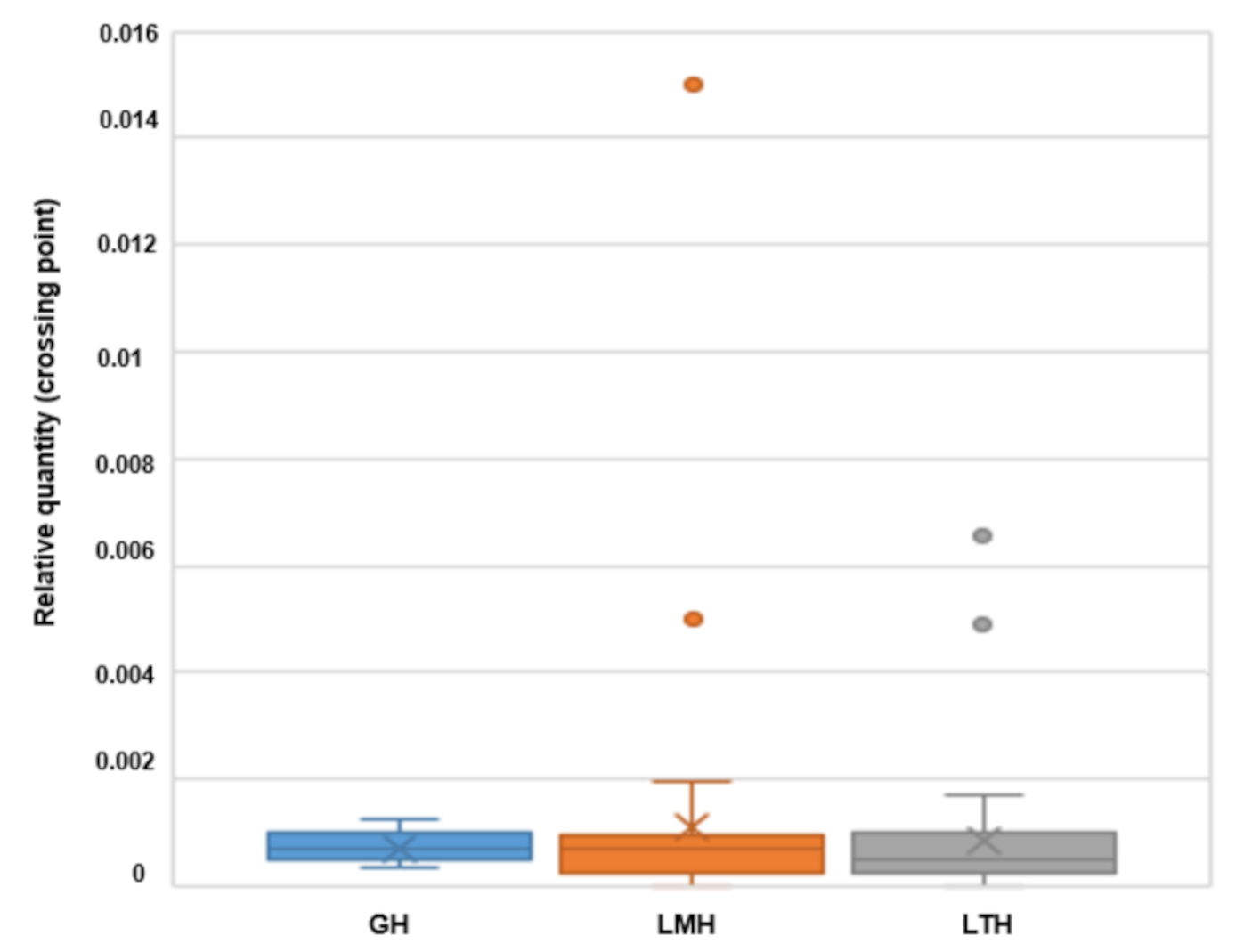

Supplement: S5 Fig — (TIF) [file pntd.0012458.s009.tif]
